# Association of *PNPLA3 rs738409* and *TM6SF2 rs58542926* with health services utilization in a population-based study

**DOI:** 10.1186/s12913-016-1289-6

**Published:** 2016-02-03

**Authors:** Julia Köpp, Steffen Fleßa, Wolfgang Lieb, Marcello Ricardo Paulista Markus, Alexander Teumer, Georg Homuth, Henri Wallaschofski, Paul Marschall, Henry Völzke, Sebastian Edgar Baumeister

**Affiliations:** 1 Department of Community Medicine, University Medicine Greifswald, Walther-Rathenau-Straße 48, 17475 Greifswald, Germany; 2 Department of Health Care Management, Ernst Moritz Arndt University of Greifswald, Friedrich-Loeffler-Straße 70, 17489 Greifswald, Germany; 3 Institute for Epidemiology, Christian Albrechts University, Niemannsweg 11, 24105 Kiel Kiel, Germany; 4 Department of Functional Genomics, Ernst Moritz Arndt University of Greifswald, Greifswald, Germany; 5 Institute of Clinical Chemistry and Laboratory Medicine, University Medicine Greifswald, Ferdinand-Sauerbruch-Straße, 17475 Greifswald, Germany; 6 Institute for Epidemiology and Preventive Medicine, University of Regensburg, Franz-Josef-Strauß-Allee 11, D-93053 Regensburg, Germany

**Keywords:** Genotype information, Hepatic steatosis, Costs Health Services Utilization

## Abstract

**Background:**

Hepatic steatosis confers an increased risk of metabolic and cardiovascular disease and higher health services use. Associations of the single nucleotide polymorphisms (SNP) *PNPLA3 rs738409* and *TM6SF2 rs58542926* with hepatic steatosis have recently been established. This study investigates the association between *rs738409* and *rs58542926* with health services utilization in a general population.

**Methods:**

Data of 3759 participants from Study of Health in Pomerania (SHIP), a population-based study in Germany, were obtained. The annual number of outpatient visits, hospitalization and length of hospital stay was regressed on *rs738409* and *rs58542926* and adjusted for socio-economic factors, lifestyle habits, clinical factors, and health status.

**Results:**

Minor allele homozygous subjects of *rs738409* had an increased odds of hospitalization as compared to major allele homozygous subjects (odds ratio [OR] 1.51; 95 % confidence interval [CI], 1.02 to 2.15). Heterozygous subjects did not differ from major allele homozygous subjects with respect to their odds of hospitalization. The three genotype groups of *rs738409* were similar with respect to the number of outpatient visits and inpatient days. Minor allele homozygous and heterozygous subjects of *rs58542926* had higher outpatient utilization (+53.04 % and +67.56 %, *p* < 0.05, respectively) and inpatient days than major allele homozygous subjects.

**Conclusions:**

After adjustment for several confounding factors, *PNPLA3 rs738409* and *TM6SF2 rs58542926* were associated with the number of outpatient visits, hospitalization, and inpatient days. Further studies are warranted to replicate our findings and to evaluate whether genetic data can be used to identify subjects with excess health services utilization.

## Background

Despite remarkable progress in our understanding of the genetic architecture of cardiometabolic diseases, which account for substantial direct and indirect health care costs, it is unclear whether genetic information is associated with health services utilization and costs. The speed of discovery of genetic variants for common diseases has increased rapidly, leading to a debate how this information might be useful to improve screening for and stratified treatment of common disorders. One potential application is the use of genetic data to improved identification of disease susceptibility in initially healthy individuals and to focus preventive measures on those at the highest risk of future disease [1, 2]. Similarly, the present study builds upon this work by investigating whether genetic data is related to health services utilization.

Many previous studies have tried to explain variation in health services utilization, including predictive modelling of health care costs and risk-adjustment models for health insurers, and typically include socio-demographic variables, clinical conditions or diagnoses, medication data and self-rated health status [3–5]. The role of genetic data has received little attention in the area of health services research, despite its consistent association with morbidity in some areas. For example, a strong association of *PNPLA3 rs738409*, a single nucleotide polymorphism (SNP) in the patatin-like phospholipase domain-containing 3 (PNPLA3) gene, with liver fat content, fibrosis, cirrhosis and non-alcoholic fatty liver (NAFLD)-related hepatocellular carcinoma has been reported [6–9]. Recently, *TM6SF2 rs5842926* has been associated with liver fat fraction, severity of steatosis, and fibrosis [10, 11].

Previous work suggests that hepatic steatosis might lead to several comorbidities including the metabolic syndrome [12, 13] and cardiovascular diseases [14, 15]. These conditions result from a complex interplay between genetic and environmental factors and pose a huge economic burden on health care systems. Consequently, hepatic steatosis has also been related to increased future health care costs and hospitalization [16]. Because clinical conditions associated with hepatic steatosis are producing substantial health care costs, it is reasonable to assume that genetic variants in the *PNPLA3* and *TM6SF2* genes, as promoters of liver fat, might be associated with health care utilization. We assumed different mechanisms by which these SNPs might affect health services utilization. Accordingly, we tested whether the following mediators might, at least in part, explain potential associations between SNPs and health services utilization outcomes: ultrasound hepatic steatosis, serum alanine aminotransferase, ferritin, metabolic syndrome, waist circumference, body mass index, triglycerides, high-density lipoprotein (HDL) cholesterol, blood pressure, serum glucose, and glycated hemoglobin (HbA1c) [17–21].

## Methods

### Study participants

The presented data were derived from the population-based Study of Health in Pomerania (SHIP). The study design has previously been described in more detail [22]. In brief, a multistage random sample was drawn from the population aged 20 to 79 years of West Pomerania, a north-eastern coastal region of Germany. The examinations were conducted between 1997 and 2001. From the 7008 initially sampled subjects, 6265 were eligible for the study and 4308 participated (response proportion 68.8 %). We excluded participants with positive findings for hepatitis B surface antigen or presence of anti-hepatitis C virus antibodies (*n* = 28). In addition, participants who had DNA with of insufficient genotyping quality (*n* = 227); with missing data on the hepatic ultrasound measurement (*n* = 70), missing self-reports of liver cirrhosis (*n* = 6), hospitalization (*n* = 17), smoking (*n* = 19), and missing values for ferritin (*n* = 20), creatinine (*n* = 21) and waist circumference measurements (*n* = 7) had to be excluded. The analysis sample included 3759 subjects. All participants gave written informed consent and the study was approved by the ethics committee of the University of Greifswald. Weighting was used to adjust for bias due to differences in responses, probabilities of selection, and discrepancies between data from official statistics and our samples with regard to demographic and geographical distributions [23].

### Data collection and measures

Information on socio-economic characteristics, lifestyle habits, medication use and health services utilization was collected by trained and certificated medical staff during a standardized computer-assisted personal interview. Number of outpatient visits was measured using two questions: (1) Have you visited a physician (general practitioner or specialist) in the past year? and (2) If “yes,” how often did you visit a particular physician in the past year? Subjects responded to a list of 18 different types of physicians and specialists. The analyses were restricted to general health services and excluded visits to dentists. Inpatient service was measured by asking the participants if they had been hospitalized at least once in the past 12 months and by further probing for the number of hospitalized days during the past year if they answered affirmatively [16].

All participants underwent an extensive medical examination including the collection of a blood samples and a sonographic examination of the abdomen, performed by trained and certified physicians using a 5 MHz transducer and a high resolution instrument (Vingmed VST Gateway Santa Clara, CA). The sonographers were unaware of the participant’s clinical and laboratory characteristics. A hyperechogenic pattern was defined as the presence of an ultrasonographic contrast between hepatic and renal parenchyma [16]. Hepatic steatosis was defined as the presence of a hyperechogenic liver pattern.

Educational attainment was estimated by recording years of schooling completed. Income was included as “equalized” household income (in €), applying the Luxembourg Income Study recommendation to divide the household income by the square root of the number of household members [24]. Alcohol consumption was assessed using a beverage-specific quantity-frequency measure [25]: number of days with alcohol consumption (beer, wine, spirits) and average daily alcohol consumption for such a day over the past month. Average daily consumption (in grams pure ethanol per day) was calculated by multiplying frequency and amount, using beverage-specific standard ethanol contents [25]. Study participants provided information about whether they had ever smoked cigarettes regularly (never, past only, current). Waist circumference was measured to the nearest 0.1 cm using an inelastic tape midway between the lower rib margin and the iliac crest in the horizontal plane, with the subject standing comfortably with weight distributed evenly on both feet. We used body mass index (BMI) ≥ 30 for obesity. Systolic and diastolic blood pressures were measured on the right arm of rested and seated participants using a digital blood pressure monitor (HEM-705CP, Omron Corporation, Tokyo, Japan). The second and third blood pressure measurements were averaged and used for analysis. A non-fasting venous blood sample was obtained from all study participants between 07:00 AM and 04:00 PM [26] photometrically (Hitachi 704 and 171, Roche, Mannheim, Germany). Serum ferritin levels were determined by an immunoturbidimetric assay (Cobas Micra Plus, F. Hoffmann-La Roche Ltd). Serum low-density lipoprotein (LDL) cholesterol and HDL cholesterol were precipitated and measured photometrically (Boehringer). A total cholesterol/HDL ratio ≥ 5 indicated dyslipidemia [27]. Triglycerides and glucose were determined enzymatically using reagents from Roche Diagnostics (Hitachi 717, Roche Diagnostics, Mannheim, Germany). Serum alanine aminotransferase (ALT) was measured photometrically (Hitachi 704 and 171, Roche, Mannheim, Germany) [16]. HbA1c was determined by high-performance liquid chromatography (Bio-Rad Diamat Analyzer, Munich, Germany). The creatinine concentration (Jaffé method) was determined on a Hitachi 717 (Roche Diagnostics, Mannheim, Germany). The estimated glomerular filtration rate (eGFR) was estimated according to the MDRD-formula and expressed in mL/min/1.73 m^2^ [28].

The metabolic syndrome was defined as the presence of at least three of the following five components [29]: (1) central obesity (waist circumference ≥94 cm in men and ≥80 cm in women); (2) reduced HDL: non-fasting HDL <1.03 mmol/L in men and <1.29 mmol/L in women, drug treatment for reduced HDL is an alternate indicator [30]; (3) elevated blood pressure: systolic ≥130 mmHg and/or diastolic ≥85 mmHg or antihypertensive drug treatment; (4) hypertriglyceridemia: non-fasting plasma triglycerides ≥2.3 mmol/L or drug treatment for elevated triglycerides [30]; (5) hyperglycemia: non-fasting glucose level of ≥8.0 mmol/L (≥144 mg/dL) or drug treatment of elevated glucose [30].

The definition of other comorbidities was based on self-reported physician’s diagnosis or self-reported use of medication. Medical history included a recall of physician’s diagnosis of a list of 15 chronic conditions including diabetes, myocardial infarction, angina pectoris, congestive heart failure, obesity, arthritis, osteoporosis, chronic obstructive pulmonary disease, neurological disease (such as multiple sclerosis or Parkinson’s disease), upper gastrointestinal disease (ulcer, hernia, reflux), stroke, anxiety, and depression. Comorbid health status was measured by the Functional Comorbidity Index (FCI), which is a summary measure of comorbid diseases selected and weighted according to their association with physical functioning [31].

### Genotyping

Genotyping was performed using the Human SNP 6.0 Array (Affymetrix, Santa Clara, CA, USA). Hybridisation of genomic DNA was genotyped according to the manufacturer’s standard recommendations. Genotypes were determined using the Birdseed2 clustering algorithm. For quality control purposes, several control samples where added. On the chip level, only subjects with a genotyping rate on QC probe sets (QC call rate) of at least 86 % were included. All remaining arrays had a sample call rate > 92 %. Imputation of genotypes in SHIP was performed with the software IMPUTE [32] v0.5.0 based on HapMap II CEU (rs738409) or IMPUTE v2.2.2 based on 1000Genomes v3 ALL populations reference panel (rs58542926). Because *rs738409* and *rs58542926* were not directly genotyped on the array but available in the imputed dataset, the best-guess genotypes of this SNP were used for the subsequent analyses by assigning the genotype having the highest probability after imputation to the corresponding individual. Quality score of imputation measured via observed by expected variance ratio of the genotypes was 0.96 for *rs738409* and 0.99 for *rs58542926*, where quality scores may range from 0 to 1 and a value of 1 indicates nearly perfect imputation quality.

### Statistical analyses

Categorical data were expressed as percentages; continuous data were expressed as arithmetic mean (SD). Annual numbers of outpatient visits and inpatient days, income, alcohol intake and ferritin were log-transformed and the geometric means (SD) are reported, as they followed approximately a log-normal distribution. Since the number of annual outpatient physician visits exhibited a skewed and discrete distribution, the traditional linear (least square) regression model was inappropriate [33]. Therefore a negative binomial model was used to evaluate the association of *PNPLA3 rs738409* and *TM6SF2 rs5842926* with the number of outpatient visits [33]. Transformations to rate ratios [i.e. exb(ß)] were performed, which describe the percent change in the outcome. The number of inpatient days among those with hospital stay was estimated using a zero-truncated negative binomial regression and expressed as rate ratios [33]. We examined the relation of *PNPLA3 rs738409* and *TM6SF2 rs5842926* and risk of hospitalization using a logistic regression model. Results from logistic regressions were expressed as odds ratio (OR), with corresponding 95 % confidence intervals (CI). We adjusted regression models for variables known to be correlated with health services use. Model 1 was adjusted for age, sex, education, income, alcohol intake, smoking status, and GFR. Model 2 added the FCI. Next, we investigated whether a potential association between *PNPLA3 rs738409* and *TM6SF2 rs5842926* and health services utilization might be mediated by ultrasound hepatic steatosis, serum alanine aminotransferase, ferritin, metabolic syndrome, waist circumference, body mass index, triglycerides, high-density lipoprotein (HDL) cholesterol, blood pressure, serum glucose, and HbA1c [17–21]. We therefore performed models that additionally included each of these intermediate factors (models 3 to 13 in Tables 2 and 3).

Internal validation of the models was performed using a 10-fold cross-validation [34]. The performance of the regression models was evaluated using Nagelkerke’s R^2^ [34] (all models), root mean squared error and mean prediction error (number of outpatient visits, number of inpatient days) [3], and the area under the receiver operating characteristics (AUC) curve (hospitalization) [34]. The AUC of models without and with SNPs were compared using the method of DeLong and Clark-Pearson for correlated data [35]. We reported the median of all assessments of the Nagelkerke R^2^, mean prediction error, and AUC across the 10 cross-validations [34]. We performed bootstrap validation of all regression models using 300 replications as a sensitivity analysis [36]. Because the findings of the bootstrap validation were almost identical to those from the cross-validation, we reported only the results from the cross-validation procedure. Bias-corrected 95 % confidence intervals (CI) were reported [37]. Stata 13.1 SE was used for statistical analyses (Stata Corporation, College Station, TX, USA).

## Results

### Socio-economic factors, lifestyle habits, clinical factors and comorbidities by PNPLA3 rs738409 and TM6SF2 rs58542926

The socio-economic, lifestyle and clinical characteristics according to genotypes of *PNPLA3 rs738409* and *TM6SF2 rs58542926* are shown in Table 1. Regarding *PNPLA3 rs738409*, hospitalizations during the last year and annual number of inpatient days were higher in minor allele homozygous subjects (GG) than in major allele homozygous subjects (CC). Minor allele homozygous subjects also revealed a higher prevalence of hepatic steatosis and increased ALT, were older, exhibited lower waist circumferences, suffered less frequently from angina pectoris and arthritis than major allele homozygous subjects. Both groups were similar regarding the number of annual outpatient visits, sex, level of education, income, alcohol intake, smoking, and other clinical variables.

**Table 1:** Characteristics of study participants by PNPLA3 rs738409 and TM6SF2 rs58542926

	PNPLA3 rs738409			TM6SF2 rs58542926		
	CC	CG	GG	CC	CT	TT
N (%)	2238 (59.6 %)	1347 (35.8 %)	173 (4.6 %)	3471 (80.6 %)	580 (13.5 %)	28 (0.7 %)
Annual number of outpatient visits^a^	7.5 (2.5)	7.5 (3.5)	7.5 (2.6)	4.9 (2.2)	7.3 (2.6)	7.9 (2.5)*
Hospitalization during the last year, %	14.1	14.2	19.7*	7.4	13.7	14.6
Annual number of inpatient days among subjects with hospitalization during the last year, days^a^	9.7 (2.6)	9.7 (2.3)	10.6 (2.5)*	6.9 (1.2)	9.6 (2.5)	11.1 (2.5)**
Age, years	49.91 (16.28)	49.40 (16.58)	50.00 (15.83)	49.3 (16.2)	48.7 (16.5)	47.4 (16.1)
Female, %	50.7	50.6	56.7*	50.1	54.7	74.1
School education (<10 years), %	39.1	37.7	39.1	38.4	40.3	33.3*
Equivalent household income, €^a^	852 (1.7)	833 (1.8)	879 (1.7)	852 (1.7)	807 (1.8)	929 (1.6)
Alcohol intake, g/d^a^	7.3 (3.5)	7.5 (3.4)	5.9 (2.5)**	7.5 (3.5)	6.9 (3.5)	5.1 (3.9)
Regular smoking, %	29.7	32.3	28.3	30.4	31.3	25.9
Body mass index, kg/m^2^	27.3 (4.8)	27.1 (4.8)	26.9 (4.8)	27.3 (4.5)	27.3 (4.8)	26.2 (4.3)
Obesity, %	26.8	24.1	22.0	25.3	28.1	14.8
Waist circumference, cm	90.0 (14.0)	88.4 (13.8)**	87.3 (13.3)**	89.2 (13.9)	89.2 (14.2)	84.4 (13.1)
Systolic blood pressure, mmHg	135.6 (20.7)	135.6 (20.7)	136.2 (20.8)	132.4 (18.5)	136.0 (21.2)	134.6 (20.8)
Hypertension, %	51.9	52.8	52.6	37.0	52.9	49.1
Ferritin, μg/l^a^	68.4 (2.7)	68.6 (2.6)	61.56 (2.58)	68.2 (2.6)	68.5 (2.7)	66.6 (3.0)
Hepatic steatosis, %	27.4	31.9**	39.9**	28.4	36.3**	44.4*
Increased ALT (>75th percentile), %	17.9	22.7**	28.3**	19.4	24.2*	29.6
GFR, ml.min.1.73 m^2^	79.5 (15.1)	80.0 (15.1)	79.8 (15.1)	79.5 (15.1)	80.9 (14.8)	78.8 (11.1)
High-density lipoprotein cholesterol,	1.45 (0.45)	1.46 (0.43)	1.49 (0.43)	1.45 (0.44)	1.48 (0.43)	1.50 (0.39)
Low-density cholesterol, mmol/l	3.59 (1.18)	3.54 (1.13)	3.54 (1.25)	3.59 (1.25)	3.50 (1.10)*	2.75 (1.27)**
Total cholesterol,	5.80 (1.28)	5.72 (1.19)	5.76 (1.32)	5.79 (1.25)	5.69 (1.22)**	4.82 (1.43)**
Dyslipidemia (TC/HDL-C ratio ≥ 5), %	27.8	25.8	24.3	27.6	24.0	14.8
triglycerides	1.91 (1.57)	1.80 (1.36)	1.78 (1.31)	1.89 (1.46)	1.74 (1.62)**	1.34 (1.53)**
Serum glucose	5.70 (1.87)	5.62 (1.67)	5.53 (1.76)	5.66 (1.81)	5.70 (1.81)	5.45 (1.00)
HbA1c, %	5.45 (0.97)	5.42 (0.88)	5.34 (0.84)	5.44 (0.92)	5.42 (0.99)	5.38 (0.49)
Diabetes, %	8.0	7.6	6.9	7.7	8.6	7.4
Upper gastrointestinal disease (ulcer, hernia, reflux), %	1.6	2.1	1.7	1.7	2.1	3.7
Myocardial infarction, %	3.4	3.3	3.5	3.3	3.4	3.9
Angina pectoris, %	5.0	3.4*	1.7*	3.9	5.8*	7.4
Congestive heart failure, %	12.51	10.62	14.45	22.2	12.0	11.1
Arthritis	7.2	7.3	11.6*	7.4	7.7	11.1
Osteoporosis	4.7	4.3	4.1	4.5	4.9	0
Chronic obstructive pulmonary disease, %	6.1	5.0	4.6	5.6	5.4	14.8
Multiple sclerosis or Parkinson’s disease, %	0.5	0.2	0	0.4	0.2	0
Stroke or TIA, %	2.3	1.6	1.7	2.1	1.7	0
Peripheral vascular disease, %	1.4	1.0	0.6	1.4	0	0
Degenerative disc disease, %	37.2	35.0	38.2	36.6	35.4	40.7
Anxiety, %	23.5	22.9	22.0	23.2	23.0	33.3
Depression, %	12.4	12.8	17.9*	12.6	13.9	14.8
Functional Comorbidity Index^a^	1.6 (1.7)	1.6 (1.6)	1.7 (1.7)	1.6 (1.6)	1.6 (1.6)	1.7 (1.6)

Entries are mean (SD) or % unless indicated differently

*GFR* glomerular filtration rate, *TC* total cholesterol; *HDL* High-density lipoprotein cholesterol

^a^Geometric mean (geometric SD)

*p < .05

**p < .01 for comparisons with CC-allele from *t*-test (continuous variables) or Fisher’s exact test (categorical variables)

Furthermore, comparing the heterozygous subjects (CG) with major allele homozygous subjects, heterozygous subjects more frequently exhibited hepatic steatosis and increased ALT and had a lower waist circumference. Both groups did not differ regarding the probability of hospitalization during the last year, the annual number of inpatient days as well as other socio-economic, lifestyle, and clinical factors (Table 1). Minor allele homozygous subjects (TT) of TM6SF2 rs58542926 more often had hepatic steatosis and more annual outpatient and inpatient days than major allele homozygous subjects (CC). Minor allele homozygous subject also had lower educational attainment, higher ferritin, lower LDL and total cholesterol, and lower triglyceride levels. Heterozygous subjects (CT) of TM6SF2 rs58542926 more frequently hepatic steatosis, increases ALT, lower LDL cholesterol, lower total cholesterol, and higher triglycerides than major allele homozygous subjects.

### Regression analyses of PNPLA3 rs738409and TM6SF2 rs58542926 with outpatient services utilization and hospitalization

Results of regression models for the association between *PNPLA3 rs738409* and health services utilization and hospitalization are summarized in Table 2. Logistic regression analysis revealed that minor compared to major allele homozygous subjects had 1.51 higher odds (95 %-CI: 1.02–2.15) of hospitalization after adjustment for age, sex, education, income, alcohol intake, smoking status, waist circumference, and GFR (Table 2, model 1). Further adjustment for comorbid conditions did not attenuate the OR (Table 2, model 2). In contrast, genotype groups did not differ with regard to the numbers of annual outpatient and inpatient visits, after full adjustment. In addition, heterozygous subjects did not differ from major allele homozygous subjects regarding the annual number of outpatient visits, odds of hospitalization, and the annual number of inpatient days.

**Table 2:** Associations of *PNPLA3 rs738409* with health utilization and hospitalization (*n* = 3759)

Reference: *PNPLA3 rs738409 CC*	Effect on annual outpatient visits, % Bootstrap (95 %-CI)^a^	Hospitalization, OR Bootstrap (95 %-CI)	Effect on annual inpatient days, % Bootstrap (95 %-CI)^a^
Model 1 (age, sex, school education, income, alcohol intake, smoking status, GFR)			
CG	−3.17 (−9.00, +4.08)	1.01 (0.83, 1.21)	−9.00 (−25.12, +7.10)
GG	+2.93 (−12.9, +21.7)	1.51 (1.02, 2.15)*	+7.38 (−22.45, +42.87)
Model 2: model 1 + Functional Comorbidity Index			
CG	−2.43 (−8.5, +3.5)	1.03 (0.89, 1.23)	−10.31 (−23.1, +10.44)
GG	+1.81 (−11.62, +18.29)	1.51 (1.01, 2.28)*	+7.49 (−21.24, +48.61)
Model 3: Model 2 + hepatic steatosis			
CG	−2.64 (−8.89, +2.65)	1.03 (0.83, 1.23)	−11.30 (−26.33, +8.15)
GG	+1.98 (−9.49, +19.92)	1.54 (1.03, 2.17)*	+7.51 (−23.45, +59.25)
Model 4: Model 2 + ALT			
CG	−2.33 (−8.82, +4.83)	1.04 (0.85, 1.26)	−9.90 (−23.65, +3.81)
GG	+2.84 (−9.20, +21.91)	1.56 (1.02, 2.27)*	+8.80 (−22.86, 50.62)
Model 5: Model 2 + ferritin			
CG	−2.41 (−7.74, +5.52)	1.02 (0.85, 1.20)	−11.3 (−25.02, +7.89)
GG	+1.57 (−12.67, +25.28)	1.49 (1.01, 2.35)*	+7.42 (−67.07, +49.20)
Model 6: Model 1 + metabolic syndrome			
CG	−2.45 (−8.97, +4.41)	1.03 (0.84, 1.31)	−9.73 (−24.1, +10.60)
GG	+4.13 (−9.85, +24.94)	1.53 (1.02, 2.28)*	+6.23 (−24.49, +40.60)
Model 7: Model 1 + waist circumference			
CG	−2.48 (−9.04, +4.56)	1.02 (0.84, 1.23)	−9.34 (−25.14, +7.67)
GG	+4.18 (−9.17, +20.31)	1.51 (1.02, 2.26)*	+7.29 (−22.41, +50.45)
Model 8: Model 1 + BMI			
CG	−2.74 (−8.65, +4.67)	1.02 (0.85, 1.22)	−9.44 (−23.11, +8.88)
GG	+3.31 (−14.11, +20.54)	1.51 (0.99, 2.23)*	+6.75 (−28.51, +43.60)
Model 9: Model 1 + triglycerides			
PNPLA3			
CG	−2.70 (−8.43, +5.48)	1.02 (0.87, 1.31)	−7.83 (−23.36, +10.71)
GG	+3.16 (−11.65, +23.22)	1.51 (1.01, 2.21)*	+7.02 (+22.9, +58.96)
Model 10: Model 1 + HDL cholesterol			
CG	−2.83 (−9.55, +3.89)	1.005 (0.83, 1.25)	−9.52 (−23.12, +7.25)
GG	+3.24 (−7.22, +23.89)	1.52 (1.02, 2.22)*	+7.97 (−27.34, +44.13)
Model 11: Model 1 + systolic blood pressure			
CG	−2.69 (−8.49, +3.44)	1.02 (0.85, 1.24)	−9.03 (+26.12, +10.44)
GG	+3.44 (−14.57, +19.68)	1.52 (1.03, 2.32)*	+7.02 (−22.73, +43.84)
Model 12: Model 1 + serum glucose			
CG	−2.92 (−9.10, +4.59)	1.02 (0.83, 1.22)	−9.59 (−24.54, +12.97)
GG	+3.30 (+11.4, +23.1)	1.49 (1.02, 2.24)*	+6.06 (−24.05, 43.55)
Model 13: Model 1 + hbA1c			
CG	−3.32 (−9.67, +4.24)	1.02 (0.83, 1.22)	−9.20 (−23.78, +7.61)
GG	+4.44 (−11.01, +26.81)	1.52 (0.96, 2.05)*	+7.79 (−21.70, +41.90)

Model 1 included age, sex, school education, income, alcohol intake, smoking status, eGFR

Bias-corrected Bootstrap confidence intervals

*CI* confidence interval

^a^Percent change compared to the ‘CC-genotype’ group

*p -value < 0.05

We assumed that the association between *PNPLA3 rs738409* and health services utilization might be mediated by hepatic steatosis, ALT, ferritin, the metabolic syndrome, waist circumference, BMI, triglycerides, HDL, systolic blood pressure, serum glucose or hbA1c (Table 3, models 3 to 13). We found that the point estimates for the association of *PNPLA3 rs738409* with health services utilization were largely unchanged after adjustment for any of these hypothesized mediators (except for serum glucose), which provides little evidence that these measured phenotypes are intermediate steps on the causal pathway from *PNPLA3 rs738409* to hospitalization.

**Table 3:** Associations of *TM6SF2 rs58542926* with health utilization and hospitalization (*n* = 3759)

Reference: TM6SF2 rs58542926 *CC*	Effect on annual outpatient visits, % Bootstrap (95 %-CI),^a^	Hospitalization, OR Bootstrap (95 %-CI)	Effect on annual inpatient days, % Bootstrap (95 %-CI),^a^
Model 1 (age, sex, school education, income, alcohol intake, smoking status, eGFR)			
CT	+53.04 (+18.33, +115.29)*	1.93 (0.67, 5.03)	+85.33 (+18.94, +188.78)*
TT	+67.56 (+28.28, +134.59)*	1.81 (0.59, 5.08)	+123.74 (+33.14, +275.97)*
Model 2: model 1 + Functional Comorbidity Index			
CT	+57.82 (+23.62, +113.43)*	1.96 (0.68, 5.56)	+94.01 (37.83, 173.1)*
TT	+69.79 (+31.60, 145.43)*	1.82 (0.72, 5.75)	+133.49 (51.86, 259.0)*
Model 3: Model 2 + hepatic steatosis			
CT	+56.54 (+15.03, +110.21)*	1.93 (0.78, 5.44)	+93.36 (+35.99, +174.93)*
TT	+69.28 (+24.58, +130.01)*	1.80 (0.68, 5.10)	+132.83 (+51.83, +257.1)*
Model 4: Model 2 + ALT			
CT	+56.51 (+19.96, +112.33)*	1.91 (0.65, 4.98)	+91.50 (+40.32, +161.36)*
TT	+69.65 (+31.38, 136.92)*	1.79 (0.63, 5.04)	+131.08 (+53.60, 247.63)*
Model 5: Model 2 + ferritin			
CT	+55.75 (+21.37, +106.99)*	1.83 (0.63, 5.38)	+94.00 (+37.65, +173.43)*
TT	+69.09 (+31.92, +134.08)*	1.73 (0.56, 4.98)	+133.47 (+52.00, +259.61)*
Model 6: Model 1 + metabolic syndrome			
CT	+45.21 (+7.26, +107.45)**	1.81 (0.65, 5.34)	+74.58 (+13.38, +168.79)**
TT	+60.48 (+14.54, 124.9)*	1.73 (0.68, 5.63)	+114.92 (+30.05, +255.17)*
Model 7: Model 1 + waist circumference			
CT	+49.27 (7.63, +95.57)*	1.92 (0.80, 5.12)	+90.50 (+32.88, +73.10)*
TT	+63.27 (+23.38, +138.34)*	1.79 (0.70, 4.71)	+131.9 (+47.56, 264.6)*
Model 8: Model 1 + BMI			
CT	+50.28 (+12.46, 106.67)*	1.92 (0.58, 5.49)	+92.70 (+27.32, 191.65)*
TT	+64.73 (+23.39, +119.28)*	1.80 (0.55, 5.21)	+133.3 (+41.87, 283.35)*
Model 9: Model 1 + triglycerides			
CT	+50.08 (+15.38, +195.16)*	1.89 (0.68, 5.61)	+83.47 (+16.35, +189.29)*
TT	+65.92 (+23.72, +120.61)*	1.79 (0.62, 5.14)	+119.17 (+29.87, 269.89)*
Model 10: Model 1 + HDL cholesterol			
CT	+50.69 (+15.98, +238.0)*	1.89 (0.69, 5.14)	+77.12 (+11.73, +180.78)**
TT	+66.26 (+26.23, +158.59)*	1.75 (0.64, 5.04)	+116.1 (+27.42, +266.50)*
Model 11: Model 1 + systolic blood pressure			
CT	+53.16 (+22.27, +141.1)*	1.93 (0.60, 5.09)	+84.63 (+18.88, +186.75)*
TT	+66.64 (+30.17, 167.93)*	1.77 (0.51, 4.73)	+124.11 (+33.36, +276.62)*
Model 12: Model 1 + serum glucose			
CT	+50.51 (+13.57, +105.38)*	1.91 (0.64, 5.50)	+82.57 (+17.68, 183.2)*
TT	+63.36 (+21.72, 116.9)*	1.81 (0.58, 5.47)	+121.82 (+32.44, 271.50)*
Model 13: Model 1 + hbA1c			
CT	+53.38 (+21.00, +109.69)*	1.94 (0.82, 5.74)	+85.09 (+18.75, +188.49)*
TT	+67.02 (+29.44, 23.17)*	1.82 (0.72, 5.52)	+122.18 (+32.83, 275.01)*

Model 1 included age, sex, school education, income, alcohol intake, smoking status, eGFR

Bias-corrected Bootstrap confidence intervals

*CI* confidence interval

^a^ Percent change compared to the ‘CC-genotype’ group

*p-value < 0.01

**p-value < 0.05

Next, we regressed health services utilization outcomes on *TM6SF2 rs58542926* (Table 3). Heterozygous subjects (CT) and minor homozygotes (TT) had an approximately 53 % (95 % CI: 18.3–67.6) and 68 % (95 % CI: 28.3–134.6) higher number of annual outpatient visits then major homozygotes after adjustment for age, sex, education, income, alcohol, and GFR (Table 3, model 1). Further adjustment for comorbid conditions slightly increased effect sizes (Table 3, model 2). To evaluate mediation, we tested whether including assumed intermediate variables change the effect sizes (Table 3, model 3 to 13). Point estimates were slightly attenuated after adding the metabolic syndrome, waist circumference, triglycerides and serum glucose to model 1. Likewise, *TM6SF2 rs58542926* was an independent predictor of length of hospital stay. Minor heterozygous subjects had a 123 % (95 %-CI: 33.1–276.0) and heterozygous subjects a 85 % (95 % CI: 18.9–188.8) higher number of annual hospital days than major homozygotes (Table 3, model 1). Inclusion of metabolic syndrome and its single components attenuated point and variance estimates (Table 3, models 3 to 13).

### Incremental predictive power of PNPLA3 rs738409and TM6SF2 rs58542926

To examine the incremental predictive power for prediction of annual outpatient visits and correct for optimistic prediction, we conducted cross-validation of all regression models. We computed measures of predictive performance for model 2, model 2 plus each of the SNPs, and model 2 plus both SNPs (Table 4). Adding both SNPs to model 2 resulted in small changes in the R^2^ values, mean squared errors, absolute prediction error, and AUC.

**Table 4:** Predictive power of models with established health utilization predictors without and with SNPs: results of the 10-fold cross validation

Statistical evaluation criteria	Annual outpatient visits	Hospitalization	Annual inpatient days
Median (Nagelkerke R^2^)			
Model 2	22.62	1.61	0.0175
Model 2 + *PNPLA3 rs738409*	22.96	1.74	0.0138
Model 2 + *TM6SF2 rs58542926*	20.33	1.56	0.0218
Model 2 + *PNPLA3 rs738409 + TM6SF2 rs58542926*	22.73	1.99	0.0268
Median (root mean squared error)		–	
Model 2	8.70		8.111
Model 2 + *PNPLA3 rs738409*	8.78		7.121
Model 2 + *TM6SF2 rs58542926*	8.69		7.422
Model 2 + *PNPLA3 rs738409 + TM6SF2 rs58542926*	8.68		7.174
Median (absolute prediction error)		–	
Model 2	5.89		3.605
Model 2 + *PNPLA3 rs738409*	5.97		3.548
Model 2 + *TM6SF2 rs58542926*	6.01		3.565
Model 2 + *PNPLA3 rs738409 + TM6SF2 rs58542926*	6.07		3.594
Area under ROC curve (*p*-value for comparison to model 2)	–		–
Model 2		0.605	
Model 2 + *PNPLA3 rs738409*		0.605 (0.846)	
Model 2 + *TM6SF2 rs58542926*		0.606 (0.868)	
Model 2 + *PNPLA3 rs738409 + TM6SF2 rs58542926*		0.606 (0.965)	

Model 2: age, sex, school education, income, alcohol intake, smoking status, eGFR, Functional Comorbidity Index

*ROC* receiver operating characteristic

## Discussion

Previous studies have reported a consistent association of *PNPLA3 rs738409* and *TM6SF2 rs58542926* with hepatic steatosis [7–11, 38, 39], which results in increased future health care expenditures [16]. Accordingly, we hypothesized that *PNPLA3 rs738409* or *TM6SF2 rs58542926* might be useful to identify subjects who would have increased health services utilization. Therefore, we correlated *PNPLA3 rs738409* and *TM6SF2 rs58542926* directly with different measures of health services utilization in a general population sample. We observed that the minor allele of *PNPLA3 rs738409* was associated with increased odds of hospitalisation. Similarly, both minor allele homozygous and heterozygous subjects of *TM6SF2 rs58542926* exhibited a higher number of annual outpatient visits and more hospital days. We also investigated mediation by variables related to fatty liver disease and components of the metabolic syndrome. The regression coefficients of *PNPLA3 rs738409* and *TM6SF2 rs58542926* were essentially unchanged upon adjustment for measured hepatic steatosis, ALT or ferritin but attenuated after inclusion of features of the metabolic syndrome. This indicates that components of the metabolic syndrome might be intermediate variables on the pathway from *PNPLA3 rs738409* and *TM6SF2 rs58542926* to health services utilization. Inclusion of the SNPs only marginally improved the predictive performance of models predicting the number of outpatient visits, hospitalization, and the number of inpatient days.

The reasons for the significant association of *PNPLA3 rs738409* and *TM6SF2 rs58542926* with outpatient and inpatient services utilization even after controlling for multiple covariables are not entirely clear. One possible explanation is that SNPs are associated with unmeasured phenotypes that cause health care outcomes that were not included in our regression models. Some assessments of chronic diseases were based on self-report data from our standardized interview, which is less specific and might result in misclassification further increasing the likelihood of detecting false-positive association. Another possible explanation is that *PNPLA3 rs738409* and *TM6SF2 rs58542926* is not directly responsible for the association but is in linkage disequilibrium with another SNP, which might have introduced genetic confounding.

As far as we know, this is the first population-based study that uses genetic data as correlates of health services utilization and investigates whether adding genetic information to a predictive model of established health services predictors improves prediction. The findings gave some indications that individual genetic data might be useful in screening for excess health services utilization and costs. Access to health care is unrestricted in Germany and cost of treatment is either covered by private or statutory health insurance. Screening based on genetic information has not been introduced in Germany. However, for making decisions regarding genetic testing it has to be considered that the inclusion of genotype information in our multivariable models with established predictors of health care utilization did not improve prediction and an external replication of our findings needs to be conducted to support our data.

The ethical and economic implications of genetic screening need to be examined before any recommendations can be made [40]. False-positive screening results can lead to additional diagnostic tests or cause unnecessary anxiety and thereby needless increase in health care use [41]. It is essential that genetic screening and its consequences are transparent and adequately understood by the target population [42, 43]. A serious critical ethical issue of genetic testing is the potential for discrimination and stigmatisation of individuals and groups. To assess genetic screening, economic evaluations need to be performed [44].

Our study has several strengths and limitations that need to be considered. Major strengths of this study include the sample size, the comprehensive clinical characterization, strict quality control procedures and standardized protocol as well as trained and certified staff for all data acquisitions. However, due to the non-fasting status of our participants a non-standard definition of the metabolic syndrome had to be applied [30] and it is not clear how this could have affected our estimates. Health care utilization outcomes were assessed using questionnaire self-reports. The usual limitations of this approach apply, especially the underreporting in self-reports and incomplete assessment of services. However, relative effects, which are of primary interest in this study, may be less biased than absolute numbers. Claims data containing the direct medical cost would help to improve the validity of the findings. Furthermore, the study was performed in a Caucasian, European population. This is not clear whether the observed findings are generalizable to other populations. Subjects that refused to take part in the study might have been different from participants with regard to health services utilization and their genetic profile, which might have introduced selection bias. Another possible limitation of this study is the imputation of SNPs and the lack of information about the specific causes of hospitalization.

## Conclusions

In conclusion, the study illustrated that genetic information might be associated with health services utilization. Further studies in independent cohorts are needed to replicate our findings.

## Abbreviations

AUC: area under the receiver operator curve
BIC: Bayesian Information Criterion
CC: major allele homozygous subjects
CG: heterozygous subjects
CI: confidence interval
FCI: Functional Comorbidity Index
GFR: glomerular filtration rate
GG: minor allele homozygous subjects
HDL-C: high-density lipoprotein cholesterol
OR: odds ratio
PNPLA3: patatin-like phospholipase domain-containing 3
SHIP: Study of Health in Pomerania
SNP: Single Nucleotide Polymorphism
TC: total cholesterol
